# Supplementary material for: Analysis of Chemical Constituents of Chrysanthemum morifolium Extract and Its Effect on Postprandial Lipid Metabolism in Healthy Adults
Source: Molecules. 2023 Jan 6;28(2):579. doi: 10.3390/molecules28020579 (PMC9866508; doi:10.3390/molecules28020579)
Supplement: Supplementary file 1 [file molecules-28-00579-s001.zip › Table S1.pdf]

Table S1. Representative differential metabolites of IC extracts

Compound	Molecular mass	Molecular formula	q1(m/z)	q3(m/z)	P-value	Q-value	RSD
**Amino acid and derivatives**							
up-regulated							
N-γ-Acetyl-N-2-Formyl-5-methoxykynurenamine	264.11	$C_{13}H_{16}N_2O_4$	265.1	136.1	0.029765863	0.077277311	0.32
Phenylacetylglycine	193.07	$C_{10}H_{11}NO_3$	194.1	91.1	0.036602785	0.086814102	0.61
L-Phenylalanine	165.08	$C_9H_{11}NO_2$	166.1	120.1	0.008186012	0.042276587	0.17
down-regulated							
4-Aminobutyric acid	103.06	C4H9NO2	104.1	87.1	0.01042725	0.046108839	0.14
5-Aminovaleric acid	117.08	C5H11NO2	118.1	55.1	0.010049308	0.045541768	0.16
5-oxoproline	129.04	C5H7NO3	152.0	69.0	0.001572898	0.017906783	0.32
cis-4-Hydroxy-D-proline	131.06	C5H9NO3	130.1	45.0	0.003589025	0.02827341	0.18
Glutathione	307.08	C10H17N3O6S	308.1	76.0	0.000534449	0.014491981	0.11
Guanidineacetic acid	117.05	C3H7N3O2	118.1	43.0	0.036751357	0.087007227	0.11
L-Asparagine	132.05	C4H8N2O3	133.1	74.0	0.000287076	0.01277121	0.11
L-Aspartic acid	133.04	C4H7NO4	134.0	74.0	0.000887443	0.015453988	0.11
L-Glutamic acid	147.05	C5H9NO4	148.1	84.0	0.000184158	0.011214295	0.04
L-Histidine	155.07	C6H9N3O2	156.1	110.1	0.001893597	0.019136272	0.06
L-Homoglutamic acid	161.07	C6H11NO4	162.1	98.1	0.023481512	0.070278636	0.12
L-Homoserine	119.06	C4H9NO3	120.1	56.0	0.037244336	0.087643013	0.07
L-Glutamine	146.11	C6H14N2O2	147.1	84.1	0.003329536	0.027030839	0.17
L-Ornithine	132.09	C5H12N2O2	133.1	70.1	0.001555497	0.017852199	0.08
L-Pipecolic acid	129.08	C6H11NO2	130.1	84.1	0.015752037	0.058057921	0.16
(2E)-Decenoyl-ACP	129.08	C6H11NO2	130.1	84.1	0.000284298	0.012740282	0.08
L-Serine	105.04	C3H7NO3	106.0	60.0	0.021543143	0.067666922	0.14
L-Threonine	119.06	C4H9NO3	120.1	56.1	0.037244336	0.087643013	0.07
trans-4-Hydroxy-L-proline	131.06	C5H9NO3	132.1	86.1	0.007922942	0.041740673	0.19
**Phenol**							
up-regulated							
1,7-Diphenyl-4-hepten-3-one	264.15	C19H20O	265.2	105.1	0.029368187	0.076889893	0.22
2,3-Dihydroxybenzoic acid	154.03	C7H6O4	153	109	0.028786022	0.076310775	0.28
3,4-Dihydroxybenzaldehyde	138.03	C7H6O3	139	65	0.015950404	0.058441868	0.20
Benzaldehyde	106.04	C7H6O	107	79	0.015141371	0.056846782	0.18
Ginkgolic acid C17:1	374.28	C24H38O3	375.3	331.3	0.000110941	0.009214895	0.10
Metanephrine	197.11	C10H15NO3	198.1	180.1	0.028536031	0.076057611	0.39
Phloretic acid	166.06	C9H10O3	167.1	121.0666667	0.020123221	0.065583609	0.20
Protocatechuic acid	154.03	C7H6O4	153.05	108.95	0.022832404	0.069431904	0.19
Salicylic acid	138.03	C7H6O3	137	93	0.029647168	0.07716236	0.18
Vanillic acid	168.04	C8H8O4	169	65	0.008921659	0.043668935	0.17
Zingerone	194.09	C11H14O3	195.1	137.1	0.001924049	0.019241235	0.08

Compound	Molecular mass	Molecular formula	q1(m/z)	q3(m/z)	P-value	Q-value	RSD
**Alkaloids**							
up-regulated							
3-Methylindole	131.07	C9H9N	132.1	130.1	0.007297701	0.040376141	0.17
Acarbose	645.25	C25H43NO18	646.3	163.1	0.040320143	0.091443052	0.23
Anatabine	160.10	C10H12N2	161.1	144.1	2.43805E-06	0.001182076	0.03
Baptifoline	260.15	C15H20N2O2	261.2	243.1	0.002772839	0.024090849	0.09
Caffeine	194.08	C8H10N4O2	195.1	138.1	0.022017932	0.068330251	0.25
Calystegine A3	159.09	C7H13NO3	160.1	142.1	0.019550705	0.064698611	0.24
Crotanecine	171.09	C8H13NO3	172.1	154.1	0.039193109	0.090083041	0.35
Ecgonine	185.11	C9H15NO3	186.1	168.1	0.000940638	0.015542328	0.15
Gentioflavin	193.07	C10H11NO3	194.1	148.1	0.001134631	0.016196845	0.12
Hordenine	165.12	C10H15NO	166.1	121.1	0.016990739	0.060383452	0.17
Isoquinoline	129.06	C9H7N	130.1	103.1	0.032923783	0.081795649	0.31
Macamide B	345.30	C23H39NO	346.3	91.1	0.027534244	0.075014995	0.35
Otonecine	185.11	C9H15NO3	186.1	168.1	0.000906918	0.015487414	0.15
Pilocarpine	208.12	C11H16N2O2	209.1	95.1	0.038993834	0.089838756	0.29
Tryptophan	204.09	C11H12N2O2	205.1	188.1	0.000115396	0.009324844	0.05
down-regulated							
1H-Indole-3-carboxylic acid	161.05	C9H7NO2	160	116	0.003318287	0.026975255	0.85
D-Aspartic acid	133.04	C4H7NO4	134	74	0.000887443	0.015453988	0.11
D-Serine	105.04	C3H7NO3	106	60	0.021543143	0.067666922	0.14
DL-Alanine	89.05	C3H7NO2	90.1	44	0.001007043	0.015640682	0.14
Glycine	75.03	C2H5NO2	76	30	0.017198433	0.060757185	0.11
**Carbohydrates**							
up-regulated							
1-O-Caffeoylglucose	342.10	C15H18O9	343.1	163	0.049293182	0.103983283	0.20
D-Ribose	150.05	C5H10O5	149	59	0.007785928	0.041452928	0.09
D-Xylulose	150.05	C5H10O5	133.1	43	0.040198326	0.091297796	0.06
Glucosamine	179.08	C6H13NO5	180.1	162.1	0.040803019	0.092014757	0.28
L-Gulose	180.06	C6H12O6	203.1	81.1	0.033648656	0.08282178	0.24
Sucrose	342.12	C12H22O11	365.1	203.1	0.002868988	0.024627748	0.11
down-regulated							
2-Deoxyribose 5'-phosphate	214.02	C5H11O7P	237	81	0.029452322	0.076972403	0.20
D-glucoronic acid	194.04	C6H10O7	193	59	0.004218639	0.031002546	0.34
D-Glucose 6-phosphate	260.03	C6H13O9P	259	97	0.00099313	0.015621065	0.20
Gluconic acid	196.06	C6H12O7	195.1	75	0.016475659	0.059436956	0.24
N-Acetyl-D-glucosamine	221.09	C8H15NO6	220.1	59	0.00159384	0.017971324	0.13
**Organic acids and derivatives**							

down-regulated							
Lecanoric acid	318.07	C16H14O7	317.1	167	0.005605892	0.035889358	0.17
1,2-Dimethoxybenzene	138.07	C8H10O2	139.1	51	0.030052015	0.077552085	0.24
4-Hydroxybenzoic acid	138.03	C7H6O3	137	93	0.009511277	0.044684099	0.25
**Flavonoids**							
up-regulated							
4',5,6,7-Tetramethoxyflavone	342.11	C19H18O6	343.1	107	0.048978713	0.103567608	0.53
Apiin	564.15	C26H28O14	565.5	432.9	0.001197243	0.016492431	0.09
Eriocitrin	596.17	C27H32O15	597.2	271.1	0.03838077	0.089079859	0.32
Homoorientin	448.10	C21H20O11	449	430.9	0.045406847	0.098702405	0.38
Hydrangenol	256.07	C15H12O4	257.1	105	0.025538507	0.072793727	0.34
Isovitexin 2''-O-arabinoside	564.15	C26H28O14	565.2	415.1	0.001031243	0.015673652	0.07
Kaempferol-3-O-rutinoside	594.16	C27H30O15	595	449	0.021100728	0.067034188	0.25
Karanjin	292.07	C18H12O4	293.1	105	0.008695766	0.043257119	0.31
Linarin	592.18	C28H32O14	593.2	285.1	0.00118076	0.016416639	0.12
Luteolin-6-C-glucoside	448.10	C21H20O11	449.1	431.1	0.040528307	0.091690305	0.37
Meloside A	594.16	C27H30O15	595.2	415.1	0.020890515	0.066728454	0.23
Neoastilbin	450.12	C21H22O11	451.1	123	0.043266242	0.095653954	0.11
Quercetin-3-O-sophoroside	626.15	C27H30O17	627.2	303.1	0.015457505	0.057479353	0.24
Rhoifolin	578.16	C27H30O14	579	433	0.009230188	0.044210516	0.18
Vicenin 2	594.16	C27H30O15	595.2	577.2	0.023088199	0.069768799	0.28
Vitexin 2''-glucoside	594.16	C27H30O15	595.2	415.1	0.020890515	0.066728454	0.23
		down-regulated					
Chalconaringenin	272.07	C15H12O5	272.9	152.9	0.024098525	0.071059087	0.57
Eriodictyol	288.06	C15H12O6	288.9	163	0.001575784	0.017915753	0.36
Isorhamnetin	316.06	C16H12O7	317.1	302.1	0.002015432	0.019543473	0.69
Naringenin	272.07	C15H12O5	273.3	152.8	0.006248843	0.03775125	0.44
Quercetin	302.04	C15H10O7	303	153	0.000200064	0.011525902	0.14
Quercetin-3-O-glucuronide	478.07	C21H18O13	479.1	303.1	5.9432E-06	0.001440768	0.12
Taxifolin	304.06	C15H12O7	304.8	286.8	0.001928013	0.019254737	0.45
Tricetin	302.04	C15H10O7	301	151	0.010633978	0.046449006	0.79
**Nucleotide and its derivates**							
up-regulated							
5'-Deoxyadenosine	251.10	C10H13N5O3	252.1	136.1	0.015506232	0.05757578	0.24
Cordycepin	251.10	C10H13N5O3	252.1	136.1	0.015506232	0.05757578	0.24
Deoxyguanosine	267.10	C10H13N5O4	266.1	150	0.000905132	0.015484404	0.11
Guanine	151.05	C5H5N5O	152.1	135	0.00660288	0.038689601	0.12
Thymidine	242.09	C10H14N2O5	243.1	127	0.01079042	0.046711519	0.16
Uracil	112.03	C4H4N2O2	113	70	0.036641471	0.086864458	0.22
Xanthosine	284.08	C10H12N4O6	285.1	153	0.004507231	0.032134676	0.15
down-regulated							
up-regulated							
4-Guanidinobutyric acid	145.09	C5H11N3O2	146.1	86.1	0.000491061	0.014294329	0.07
p-Toluenesulfonic acid	188.01	C7H8O4S	189	91.1	0.022832371	0.06943186	0.15
down-regulated							
Aminomalonic acid	119.02	C3H5NO4	120	74	0.019770842	0.065042106	0.20
cis-Aconitic acid	174.02	C6H6O6	175	69	0.039100836	0.089970072	0.06
Cynarin	516.13	C25H24O12	515.1	191.1	0.00174291	0.018582529	0.25
Fumaric acid	116.01	C4H4O4	115	71	0.000917867	0.015505644	0.08
Phosphoric acid	97.98	H3O4P	99	81	0.010088056	0.045601199	0.31
Shikimic acid	174.05	C7H10O5	173	93	0.028928639	0.076453984	0.13
**Fatty Acyls**							
up-regulated							
16(R)-HETE	320.24	C20H32O3	303.2	91.1	0.022353663	0.068789796	0.18
8-Methylnonenoate	170.13	C10H18O2	171.1	153.1	0.013010682	0.052243015	0.13
9-OxoODE	294.22	C18H30O3	295.2	277.2	0.013315333	0.052940104	0.19
9(S)-HPOT	310.21	C18H30O4	309.2	121.1	0.007649295	0.041159854	0.25
Eicosadienoic acid	308.27	C20H36O2	309.3	69.1	0.00734486	0.04048382	0.11
Turanose	342.12	C12H22O11	365.1	203.1	0.029662396	0.07717714	0.28
down-regulated							
Lubiprostone	390.22	C20H32F2O5	391.2	149	0.02294158	0.069576214	0.04
Pimelic acid	160.07	C7H12O4	161.1	69.1	4.70981E-05	0.006516873	0.16
**Benzene and substituted derivatives**							
up-regulated							
2-(Methylamino)benzoic acid	151.06	C8H9NO2	152.1	134.1	0.024074329	0.071028917	0.12
Benzocaine	165.08	C9H11NO2	166.1	120	0.008313089	0.042527967	0.19
Ephedrine	165.12	C10H15NO	166.1	148.1	0.049623224	0.10441741	0.37
N-Acetylarylamine	135.07	C8H9NO	134.1	92.1	0.001491827	0.017644708	0.04
Phenethylamine	121.09	C8H11N	122.1	105.1	0.004623377	0.032571466	0.12
down-regulated							
2-Hydroxy-3-(4-hydroxyphenyl) propenoic acid	180.04	C9H8O4	181	105	0.003730782	0.028921582	0.19
3-Cresotinic acid	152.05	C8H8O3	151	107.1	0.014954804	0.056467712	0.25
Sildenafil	474.20	C22H30N6O4S	473.2	282.1	0.028354177	0.07587172	0.19
Xylometazoline	244.19	C16H24N2	245.2	189.1	0.020432347	0.066050393	0.18
**Carboxylic acids and derivatives**							
up-regulated							
Isocitrate	192.03	C6H8O7	191	73	0.018707732	0.063344792	0.26
down-regulated							
D-Glutamine	146.07	C5H10N2O3	147.1	84	0.003329536	0.027030839	0.17
Malonic acid	104.01	C3H4O4	103	59	0.009731989	0.045043429	0.22
Pyrrolidonecarboxylic acid	129.042594	C5H7NO3	130	84	0.015752037	0.058057921	0.16
**Organooxygen compounds**							

Adenosine 5'-monophosphate	347.06	C10H14N5O7P	348.1	136.1	0.007633013	0.041124511	0.13
Inosine 5'-monophosphate	348.05	C10H13N4O8P	347	79	0.029810873	0.07732075	0.59
Uridine 5'-monophosphate	324.04	C9H13N2O9P	323	79	0.005913179	0.036806188	0.67
**Purine nucleosides**							
up-regulated							
2'-O-Methyladenosine	281.11	C11H15N5O4	282.1	136.1	0.000679991	0.014994232	0.07
Vidarabine	267.10	C10H13N5O4	268.1	136.1	0.017280877	0.060904305	0.26
up-regulated							
Galactinol	342.116215	C12H22O11	343.1	181.1	0.013448935	0.053241496	0.22
Lactulose	342.116215	C12H22O11	365.1	203.1	0.029662396	0.07717714	0.28
down-regulated							
Beta-D-Fructose 2-phosphate	260.03	C6H13O9P	261	99	0.005431188	0.035344387	0.14
