# Supplementary material for: Analysis of Chemical Constituents of Chrysanthemum morifolium Extract and Its Effect on Postprandial Lipid Metabolism in Healthy Adults
Source: Molecules. 2023 Jan 6;28(2):579. doi: 10.3390/molecules28020579 (PMC9866508; doi:10.3390/molecules28020579)
Supplement: Supplementary file 1 [file molecules-28-00579-s001.zip › Table S2.pdf]

Table S2. The derived index after fat loading in subjects from fasting concentrations (mean±SD).

	Control group	IC group	t	*p*		Control group	IC group	t	*p*
glucose					Apo-B				
Glu-PR	1.81±0.88	1.03±0.85	**2.75**	**0.009**	Apo-BPR	0.02±0.03	0.01±0.04	-0.05	0.96
maximal changes	2.35±1.10	1.44±0.84	**2.86**	**0.007**	maximal changes	0.06±0.02	0.06±0.04	**-1.95**	**0.05**
AUC	41.34±2.78	42.00±3.16	-0.88	0.38	AUC	5.93±1.31	5.84±1.47	0.2	0.85
IAUC	1.77±2.28	0.82±2.73	1.14	0.26	IAUC	-0.08±0.18	-0.09±0.31	-0.49	0.63
TG					T-AOC				
TGPR	0.47±0.31	0.32±0.16	1.84	0.07	T-AOCPR	0.05±0.09	-0.04±0.11	**2.73**	**0.01**
maximal changes	0.62±0.37	0.39±0.17	**2.41**	**0.024**	maximal changes	0.18±0.10	0.26±0.12	**-2.06**	**0.04**
AUC	7.20±2.53	6.21±2.19	-1.22	0.22	AUC	3.54±1.51	4.38±1.06	-1.95	0.06
IAUC	1.99±1.74	1.35±1.21	1.31	0.2	IAUC	-0.30±0.7	0.12±1.23	-0.41	0.682
HDL-C					MDA				
HDL-CPR	0.03±0.10	0.02±0.06	-0.14	0.89	MDAPR	3.65±3.78	1.20±1.96	**-2.63**	**0.009**
maximal changes	0.15±0.07	0.11±0.07	**-2.35**	**0.02**	maximal changes	6.85±4.24	3.51±2.37	**-2.75**	**0.006**
AUC	12.74±2.46	11.65±2.14	1.44	0.159	AUC	48.34±14.50	41.95±8.08	1.64	0.11
IAUC	-0.21±0.79	-0.32±0.46	-0.06	0.95	IAUC	-0.99±21.17	-2.54±12.83	-0.75	0.46
Apo-A									
Apo-APR	0.04±0.04	0.01±0.05	1.81	0.08					
maximal changes	0.11±0.04	0.10±0.04	0.35	0.73					
AUC	8.33±0.66	7.82±0.63	**2.4**	**0.02**					
IAUC	-0.15±0.26	-0.26±0.42	1	0.32					
