# Supplementary figures and images for: Analysis of Chemical Constituents of Chrysanthemum morifolium Extract and Its Effect on Postprandial Lipid Metabolism in Healthy Adults
Source: Molecules. 2023 Jan 6;28(2):579. doi: 10.3390/molecules28020579 (PMC9866508; doi:10.3390/molecules28020579)

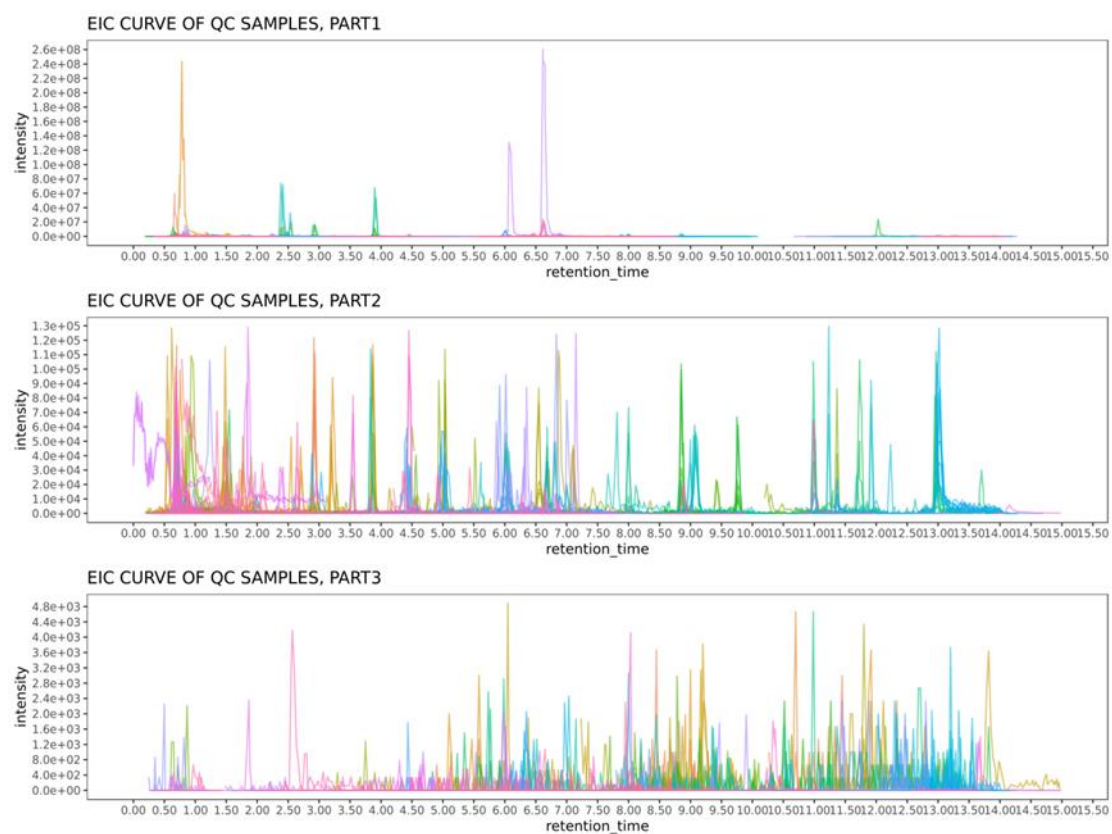

**Figure S1.** Ion current diagram of IC-E and IC-P.

Supplement: Supplementary file 1 [file molecules-28-00579-s001.zip › Figure S1.pdf]

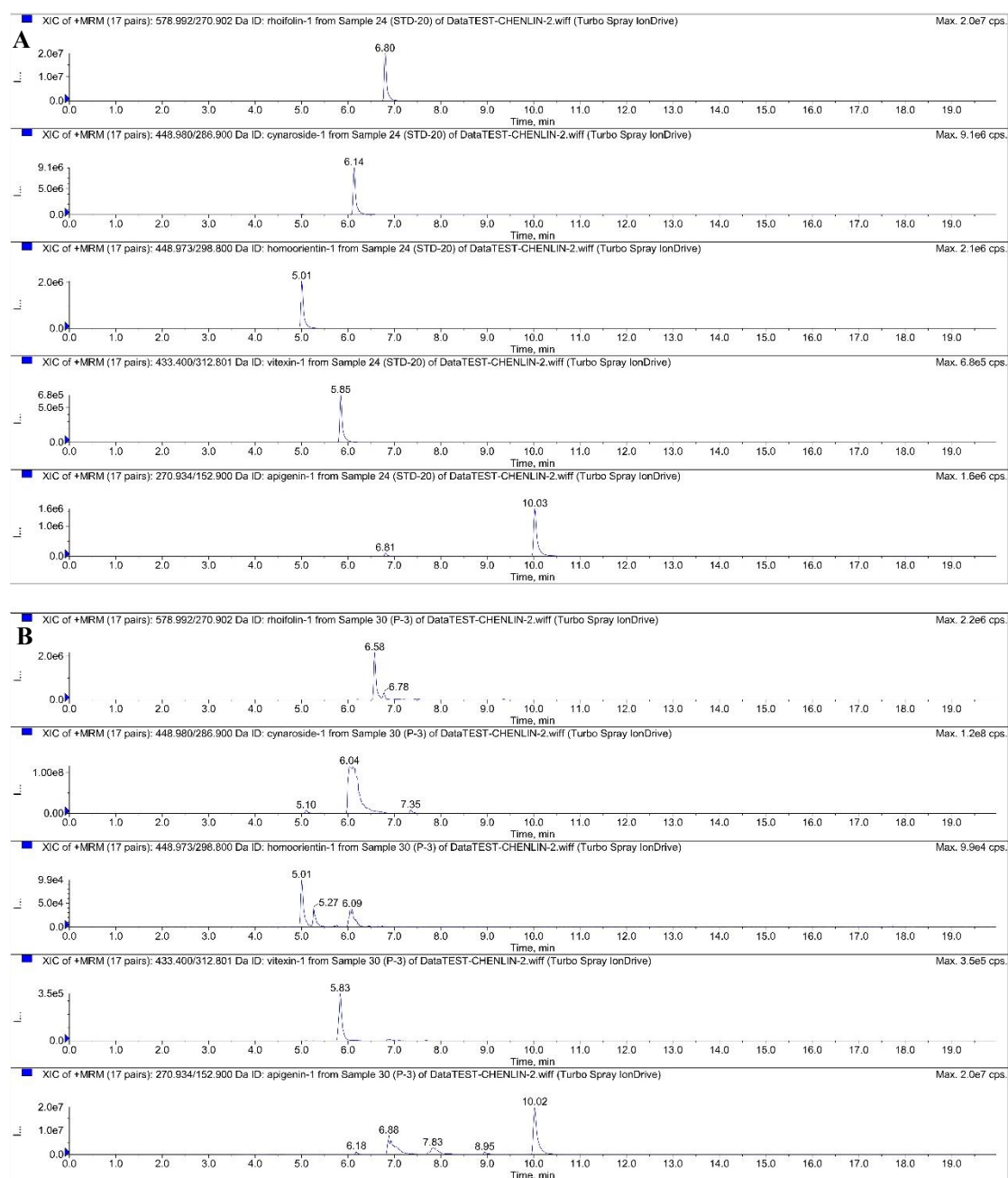

**Figure S2.** The EICs from a standard solution (A) and IC-E (B) under the optimal conditions.

Supplement: Supplementary file 1 [file molecules-28-00579-s001.zip › Figure S2.pdf]
